# miR-6881-3p contributes to diminished ovarian reserve by regulating granulosa cell apoptosis by targeting SMAD4

**DOI:** 10.1186/s12958-024-01189-8

**Published:** 2024-02-01

**Authors:** Wenhan Ju, Shuai Zhao, Haicui Wu, Yi Yu, Yuan Li, Danqi Liu, Fang Lian, Shan Xiang

**Affiliations:** 1 Shandong University of Traditional Chinese Medicine, Jinan, China; 2 Affiliated Hospital of Shandong University of Traditional Chinese Medicine, Jinan, China

**Keywords:** Apoptosis, DOR, miR-6881-3p, SMAD4

## Abstract

**Background:**

In our previous investigation, we revealed a significant increase in the expression of microRNA-6881-3p (miR-6881-3p) in follicular fluid granulosa cells (GCs) from women with diminished ovarian reserve (DOR) compared to those with normal ovarian reserve (NOR). However, the role of miR-6881-3p in the development of DOR remains poorly understood.

**Objective:**

This study aimed to elucidate the involvement of miR-6881-3p in the regulation of granulosa cells (GCs) function and the pathogenesis of DOR.

**Materials and methods:**

Initially, we assessed the expression levels of miR-6881-3p in GCs obtained from human follicular fluid in both NOR and DOR cases and explored the correlation between miR-6881-3p expression and clinical outcomes in assisted reproduction technology (ART). Bioinformatic predictions and dual-luciferase reporter assays were employed to identify the target gene of miR-6881-3p. Manipulation of miR-6881-3p expression was achieved through the transfection of KGN cells with miR-6881-3p mimics, inhibitor, and miRNA negative control (NC). Following transfection, we assessed granulosa cell apoptosis and cell cycle progression via flow cytometry and quantified target gene expression through quantitative real-time polymerase chain reaction (qRT-PCR) and Western blot (WB) analysis. Finally, we examined the correlation between target gene expression levels in GCs from NOR and DOR patients and their association with ART outcomes.

**Results:**

Our findings revealed elevated miR-6881-3p levels in GCs from DOR patients, which negatively correlated with ovarian reserve function and ART outcomes. We identified a direct binding interaction between miR-6881-3p and the 3’-untranslated region of the SMAD4. Transfection with miR-6881-3p mimics induced apoptosis in KGN cell. Furthermore, miR-6881-3p expression negatively correlated with both mRNA and protein levels of the SMAD4. The mRNA and protein levels of SMAD4 were notably reduced in GCs from DOR patients, and SMAD4 mRNA expression positively correlated with ART outcomes. In addition, the mRNA levels of FSHR, CYP11A1 were notably reduced after transfection with miR-6881-3p mimics in KGN cell, while LHCGR notably increased. The mRNA and protein levels of FSHR, CYP11A1 were notably reduced in GCs from DOR patients, while LHCGR notably increased.

**Conclusion:**

This study underscores the role of miR-6881-3p in directly targeting SMAD4 mRNA, subsequently diminishing granulosa cell viability and promoting apoptosis, and may affect steroid hormone regulation and gonadotropin signal reception in GCs. These findings contribute to our understanding of the pathogenesis of DOR.

**Supplementary Information:**

The online version contains supplementary material available at 10.1186/s12958-024-01189-8.

## Introduction

Diminished ovarian reserve (DOR) is characterized by a reduction in the quantity and quality of oocytes, typically diagnosed based on the following criteria: elevated follicle-stimulating hormone (FSH) levels (> 10 mIU/ml), a decreased antral follicle count (AFC) (< 5 to 7), or diminished anti-Mullerian hormone (AMH) levels (< 0.5 to 1.1 ng/mL) [1, 2]. While current evidence suggests that DOR may be influenced by genetics, immunity, social and environmental elements, and iatrogenic factors [3], the exact causative factors remain largely unclear. Effective pharmacological interventions for DOR are currently lacking, leading to substantial physical and emotional challenges for affected women. Even when women with DOR seek assistance through assisted reproduction technology (ART), their chances of achieving pregnancy are compromised due to limited and suboptimal oocyte availability.

Ovarian granulosa cells (GCs) are widely acknowledged to play a crucial role in nurturing oocytes by providing essential nutrients and participating in processes like oocyte maturation, ovulation, and fertilization through paracrine signaling pathways, thereby influencing oocyte quality [4]. MicroRNAs (miRNAs), a class of noncoding RNAs, regulate gene expression by interacting with the 3′‐untranslated region (3′‐UTR) of target messenger RNAs (mRNAs) [5]. In vivo, pre-miRNAs are processed by Dicer to generate mature miRNAs. Conditional knockout of the Dicer1 gene in mouse ovarian GCs has been associated with aberrant oocyte maturation, ovulatory disorders, and infertility [6]. In addition, an increasing number of studies have shown that ovarian GCs apoptosis or proliferation can be modulated by the increased or decreased expression of miRNAs (such as miR-92a [7], miR-145 [8], miR-484 [9], and miR-181 [10, 11]).

Our previous research found that there were significant differences in the expression of 70 miRNAs in follicular fluid GCs of DOR women with advanced age compared to normal ovarian reserve (NOR) women with young with significant upregulation of miR-6881-3p expression [12]. Prior investigations on miR-6881-3p have primarily centered on cancer research. Interestingly, miR-6881-3p was found to suppress autophagy in ovarian cancer by targeting ATG5 and ATG7 [13]. In hepatocellular carcinoma, miR-6881-3p was implicated in p53-mediated ceRNA networks [9]. In breast cancer, miR-6881-3p was shown to enhance the osteogenic differentiation of human adipose-derived stem cells (hASCs) [14]. However, the role of miR-6881-3p in the molecular pathogenesis of female fertility remains uncharted territory, prompting our investigation into its functions in human GCs through a combination of bioinformatics and experimental approaches.

## Materials and methods

### Clinical data analysis

#### Patient selection and preparation

Our research ethics approval was obtained from the Reproductive Medicine Ethics Committee of Affiliated Hospital of Shandong University of Traditional Chinese Medicine. In addition, all participants signed written informed consent. The study was registered with the Chinese Clinical Trial Register (Registration number: ChiCTR1800019798, https://www.chictr.org.cn/index.html).

Between January and May 2019, we selected 30 patients undergoing oocyte retrieval for in vitro fertilization (IVF) or intracytoplasmic sperm injection (ICSI) at the Center of Reproductive and Genetic, Affiliated Hospital of Shandong University of Traditional Chinese Medicine. Twenty participants were diagnosed with DOR according to the Bologna criteria (AFC < 5–7 or AMH < 0.5–1.1 ng/mL) [1], while the remaining 10 patients underwent ICSI due to male factors and served as controls. Participants were required to meet the following criteria: 1) age between 20 and 45 years old; 2) unable to conceive naturally for at least 1 year. Participants with endometriosis, hyperprolactinemia, autoimmune diseases, or any other conditions that may impact oocyte quality were excluded from the study. Participants with severe comorbidities, hematological disorders, ovarian tumors, other malignant tumors, congenital adrenal hyperplasia, and Cushing’s syndrome were excluded from the study.

#### Treatment

All patients were subjected to the antagonist protocol for ovulation induction. Basal serum reproductive endocrine hormone levels and vaginal ultrasound were examined on the second day of menstruation. If the conditions were permissive, 225–300 IU of recombinant FSH β injection (r-FSH, Purego ®, 600 IU, Merck Sharp & Dohme.) was used to initiate controlled ovarian hyperstimulation. Transvaginal ultrasound examination and serum hormone level testing were performed every 2–4 days, and the r-FSH dosage was adjusted based on follicular development. A GnRH antagonist (Ganirelix Injection, 0.25 mg, Sharp & Dohme) was administered when the leading follicle reached a 12–14 mm diameter until the trigger day. When the target follicle equal to 18 mm in diameter was formed, 250 µg recombinant human chorionic gonadotrophin (rhCG, Ovidrel™) was administered to trigger ovulation. After 34–36 h, oocyte pick‐up was performed by ultrasound‐guided transvaginal oocyte retrieval, and all follicular fluid samples were collected.

#### Isolation of GCs

The follicular fluid was collected and centrifuged at 3000 rpm/min for 10 min. Cells in the middle layer were collected with a Pasteur pipette and added to a 4 mL glass tube with PBS buffer to prepare a cell suspension. Then, the cell suspension was gently transferred to a liquid surface containing 2.5 mL of human lymphocyte separation solution (TBD). After centrifugation at 2000 rpm/min for 10 min, the GCs (the white cloud-like cell clusters) in the layer between the suspension and lymphocyte separation solution were aspirated and transferred to a 1.5 ml centrifuge tube containing PBS, mixed well, and centrifuged at 2500 rpm for 10 min. After completion, the supernatant was removed by aspiration, and the GCs were preserved in a -80°C refrigerator for further analysis.

### Bioinformatics predictions

Target genes for miR‐6881‐3p were predicted using the TargetScan (http://www.targetscan.org), DIANA (http://diana.imis.athena-innovation.gr/DianaTools/) and miRDB (http://www.mirdb.org/miRDB/) databases. Subsequently, a Venn plot was used to extract intersection target genes. To further investigate the biological functions of potential target genes, a protein-protein interaction network was established by the STRING database (https://cn.string-db.org/). In addition, the Database for Annotation, Visualization, and Integrated Discovery (DAVID) database v.6.8 was used for gene ontology (GO) analysis and the Kyoto Encyclopedia of Genes and Genomes (KEGG) pathway enrichment analysis. GO enrichment analysis includes biological processes (BP), molecular functions (MF), and cellular components (CC). Subsequently, the relative expression levels of potential target genes in various human tissues were queried in the NCBI (https://www.ncbi.nlm.nih.gov/) database. Transcription factor data were obtained through the hTFtarget (http://bioinfo.life.hust.edu.cn/hTFtarget#!/) database.

### Dual‐luciferase reporter analysis

miR‐6881‐3p mimics, miRNA negative control (NC), pmirGLO‐TGFBR1‐wild type (WT)/mutation type (MT), and pmirGLO‐SMAD4‐WT/MT vectors were synthesized by Beijing Syngenbio Co., LTD. HEK‐293T cells were cultured in Dulbecco’s modified Eagle’s medium (DMEM; HyClone) supplemented with 10% fetal bovine serum (FBS, Gibco). Then, HEK-293T cells in the logarithmic growth phase were seeded into 24‐well culture plates and transfected with the corresponding vectors when the cells attained 70%-80% confluence. Various combinations of plasmids are shown in Table 1. After 48h of transfection, luciferase activities were measured using the Dual‐Luciferase Reporter Gene Assay Kit (Biovision) following the manufacturer’s instructions.

**Table 1:** The combinations of transfected plasmids

Serial number	Plasmid combinations
1	miR-6881-3p+SMAD4 3’UTR wt
2	miR-6881-3p+SMAD4 3’UTR mt
3	miRNA NC+SMAD4 3’UTR wt
4	miRNA NC+SMAD4 3’UTR mt
5	miR-6881-3p+TGFBR1 3’UTR wt
6	miR-6881-3p+TGFBR1 3’UTR mt
7	miRNA NC+TGFBR1 3’UTR wt
8	miRNA NC+TGFBR1 3’UTR mt

### KGN cells experiment

KGN cell lines were first isolated and purified from from a patient with invasive ovarian granulosa cell carcinoma in Japan [15]. KGN cell lines have shown a pattern similar to that of steroidogenesis (the ability to synthesize estradiol and progesterone) in human granulosa cells, thus allowing analysis of naturally occurring steroidogenesis in human granulosa cells [15], which has been widely used in experiments. In our study, KGN cells were purchased from iCell Bioscience Inc (Shanghai) and cultured in a special medium (Procell, CM‐0603) containing 10% FBS. All the cells were incubated at 37℃ with 5% CO2. KGN cells were seeded into 6‐well plates at a density of 3 × 10^5^ cells per well. Transfection of miR‐6881‐3p mimics, miR‐6881‐3p inhibitor, or miRNA NC was performed using Lipofectamine 2000 (Invitrogen) when cells reached 60% confluence. After 48h of transfection, KGN cells were harvested for subsequent analysis.

#### Quantitative real‐time polymerase chain reaction

The primer sequences are shown in Table 2 for miR‐6881‐3p, U6, SMAD4, FSHR, LHCGR, CYP11A1, BCL2, BAX and β-actin. Total RNA was extracted using TRIzol (Invitrogen) and reverse-transcribed using random primers with the PrimeScript RT reagent kit (Vazyme, China). Quantitative real-time PCR(qRT-PCR) was carried out using SYBR Green qPCR Master Mix according to the manufacturer’s instructions. The relative expression of the candidate gene was normalized to the expression of the internal reference gene (β-actin for SMAD4, FSHR, LHCGR, CYP11A1, BCL2, BAX and U6 for miR‐6881‐3p) and then calculated using the 2^-ΔΔ^Ct method. All reactions were performed in triplicate.

**Table 2:** Primers used for quantitative real-time polymerase chain reaction

Gene	Primer	Primer sequence
miR-6881-3p	Primer F	GCGAGTGGGAAGGACGAAA
	Primer R	AGTGCAGGGTCCGAGGTATT
U6	Primer F	GTGCTCGCTTCGGCAGCACATAT
	Primer R	AGTGCAGGGTCCGAGGTATT
SMAD4	Primer F	GGGTCAGGTGCCTTAGTGAC
	Primer R	TGTCGATGACACTGACGCAA
FSHR	Primer F	GCTTTGAAAGTGTGATTCTATGGC
	Primer R	CAGAGGCTCCGTGGAAAACA
LHCGR	Primer F	AGGGCCGAAAACCTTACAGA
	Primer R	AGCATCTGGTTCAGGAGCAC
CYP11A1	Primer F	CTGGTGACAATGGCTGGCTA
	Primer R	TTGCCGAGCTTCTCCCTGTA
BCL2	Primer F	GGATAACGGAGGCTGGGATG
	Primer R	TGACTTCACTTGTGGCCCAG
BAX	Primer F	GGGGAGCAGCCCAGAGG
	Primer R	CGATCCTGGATGAAACCCTGA
β-actin	Primer F	CCACCATGTACCCTGGCATT
	Primer R	CGGACTCGTCATACTCCTGC

#### Western blot

Total proteins were extracted from GCs samples and KGN cells using radioimmunoprecipitation assay lysis (Boster). The protein was separated on a 12% polyacrylamide SDS-PAGE gel and transferred to a PVDF membrane. The membranes were blocked with 5% skim milk powder in TBST at room temperature for 1h while shaking. After blocking, the blots were incubated with antibodies against SMAD4 (1:1000, 10231-1-AP, Proteintech, China), FSHR (1:1000, 22665-1-AP, Proteintech, China), LHCGR (1:1500, 19968-1-AP, Proteintech, China), CYP11A1 (1:2000, 13363-1-AP, Proteintech, China), BCL2 (1:10000, 68103-1-Ig, Proteintech, China), BAX (1:8000, 50599-2-Ig, Proteintech, China), and GAPDH (1:5000, 60004-1-Ig, Proteintech, China) in Tris-buffered saline (TBS) containing 0.1% Tween-20 at 4°C overnight. The following day, the blots were incubated with goat anti-rabbit horseradish peroxidase immunoglobulin G (H + L) secondary antibody (1:5000, AS014, ABclonal, China) at 37 °C for 2h. The protein bands were analyzed by ImageJ software.

#### Flow cytometry

Cell apoptosis was evaluated by flow cytometry using the Annexin V‐FITC/PI Apoptosis Detection Kit (Beyotime, China). After digestion with trypsin, transfected cells were washed 3 times with ice-cold PBS and resuspended in 100 µl of binding buffer. Then, approximately 5 µl Annexin V-FITC (20 mg/mL) was added and incubated at 4 °C in darkness for 15 min, after which 10µl PI (50 mg/mL) (Beyotime, China) was added and incubated at 4 °C for 5 min. After adding buffer, the cells were injected into a flow cytometer to detect the apoptosis rate. The cell cycle status was determined using a PI staining assay (Sigma, USA). Briefly, the cell suspension was centrifuged for 10 min to remove the supernatant, and then the cells were resuspended in 70% ethanol at 4 °C for 12 h. The fixed cells were stained with 500 μL PI/Triton X-100 solution (0.02% RNAse, 0.002% PI, 0.1%, Triton X-100) at 37 °C for 15 min. Data were acquired using BD Accuri Cytometry (BD BioSciences, Franklin Lakes, NJ, USA).

#### Immunofluorescence staining

The cells were washed with PBS three times and then fixed with 4% paraformaldehyde for over 30 min. Then, the cells were washed with PBS thrice and permeated with 0.2% Triton X-100 at room temperature for 10 min. After the cells were washed with PBS thrice, 5% BSA solution was added and sealed at room temperature for 30 min. After sealing, the cells were incubated with the primary antibody (1:100, 10231-1-AP, Proteintech, China) at 4°C for 16h and the second antibody (1:100, SA00013-2, Proteintech, China) at room temperature for 1h. Finally, DAPI dye was added in the dark for staining. After being washed with PBS, the cells were sealed with an Antifade Mounting Medium with DAPI (Beyotime, China).

### Statistical analysis

All statistical analyses were conducted using SPSS 25.0 Statistics (IBM, USA). Descriptive statistics were presented as mean ± SD for variables with a normal distribution, and independent-sample t-tests were employed for variable description and within-group comparisons. Conversely, data with a non-normal distribution were expressed as the median (25th-75th percentile), and the Mann-Whitney test was utilized for within-group comparisons. Linear Regression analysis was applied to assess correlations between variables. A *P* value less than 0.05 was statistically significant.

## Results

### Baseline information of the participants

We collected 30 human follicular fluid samples, including 10 from individuals with NOR and 20 from those with DOR. Table 3 displays the demographic and baseline characteristics of the participants. In the DOR group, the AFC (5 [4.25, 6] vs. 24 [18.25, 24], *P* < 0.001) and AMH levels (0.32 [0.21, 0.74] vs. 4.58 [3.48, 6.95], *P* < 0.001) were significantly lower compared to the NOR group. Additionally, the basal FSH levels (15.75 [13.00, 19.54] vs. 6.08 [5.36, 7.56], *P* < 0.001) in the DOR group were significantly higher than those in the NOR group. There were no significant differences in age, duration of infertility, body mass index (BMI), basal estradiol, or basal progesterone (*P* > 0.05).

**Table 3:** Demographic and clinical characteristics of the enrolled participants

	NOR	DOR	*P*
n	10	20	
Age (year)	34.20 ± 3.71	36.65 ± 4.45	0.146
Infertile duration (year)	3.5 (1,5)	5 (2,8)	0.700
BMI (kg/m2)	22.35 ± 1.66	23.78 ± 2.04	0.052
AMH (ng/ml)	4.58 (3.48,6.95)	0.32 (0.21,0.74)	< 0.001
AFC(n)	24 (18.25,24)	5 (4.25,6)	< 0.001
Basic FSH (mIU/ml)	6.08 (5.36,7.56)	15.75 (13.00,19.54)	< 0.001
Basic LH (mIU/ml)	4.65 ± 1.20	5.32 ± 1.91	0.255
Basic estradiol (pg/ml)	32.83 (28.37,47.78)	34.06 (24.65,38.78)	0.999
Basic progesterone (ng/ml)	0.44 (0.37,0.53)	0.33 (0.22,0.41)	0.056

### The expression levels of miR-6881-3p from human follicular fluid GCs are negatively associated with ovarian reserve and ART outcomes

**Fig. 1:**
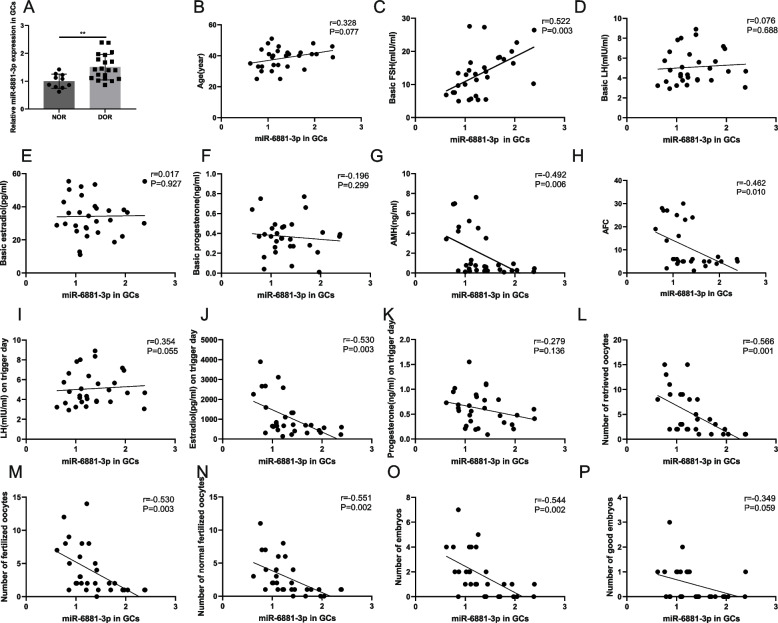
The expression levels of miR-6881-3p in human follicular fluid GCs are associated with ovarian reserve and ART outcomes. **A** The expression levels of miR-6881-3p in GCs of NOR and DOR groups; **B**-**H** Correlations between the expression levels of miR-6881-3p in GCs and age, basic FSH, basic LH, basic estradiol, basic progesterone, AMH, and AFC; **I**-**P** Correlations between the expression levels of miR-6881-3p in GCs and LH on trigger day, estradiol on trigger day, progesterone on trigger day, number of retrieved oocytes, number of fertilized oocytes, number of normal fertilized oocytes, number of embryos, and number of good embryos. ** *P* < 0.01

The results revealed that miR-6881-3p expression levels in the DOR group were significantly higher than in the NOR group (*P* < 0.01, Fig. 1A). Further analysis demonstrated a significant positive correlation between miR-6881-3p expression levels in GCs derived from human follicular fluid and basic FSH (*r* = 0.522, *P* = 0.003). Conversely, miR-6881-3p expression levels exhibited a negative correlation with AMH (*r* = -0.492, *P* = 0.006), AFC (*r* = -0.462, *P* = 0.010), estradiol on trigger day (*r* = -0.530, *P* = 0.003), number of retrieved oocytes (*r* = -0.566, *P* = 0.001), number of fertilized oocytes (*r* = -0.530, *P* = 0.003), number of normal fertilized oocytes (*r* = -0.551, *P* = 0.002), and number of embryos (*r* = -0.544, *P* = 0.002). No significant correlations were observed between miR-6881-3p levels and age, basal LH, basic estradiol, basic progesterone, LH or progesterone on trigger day, or the number of good embryos (all *P* > 0.05) (Fig. 1B-P).

### Potential effects of miR-6881-3p on ovarian granulosa cells on a transcriptome-wide scale and target gene screening

**Fig. 2:**
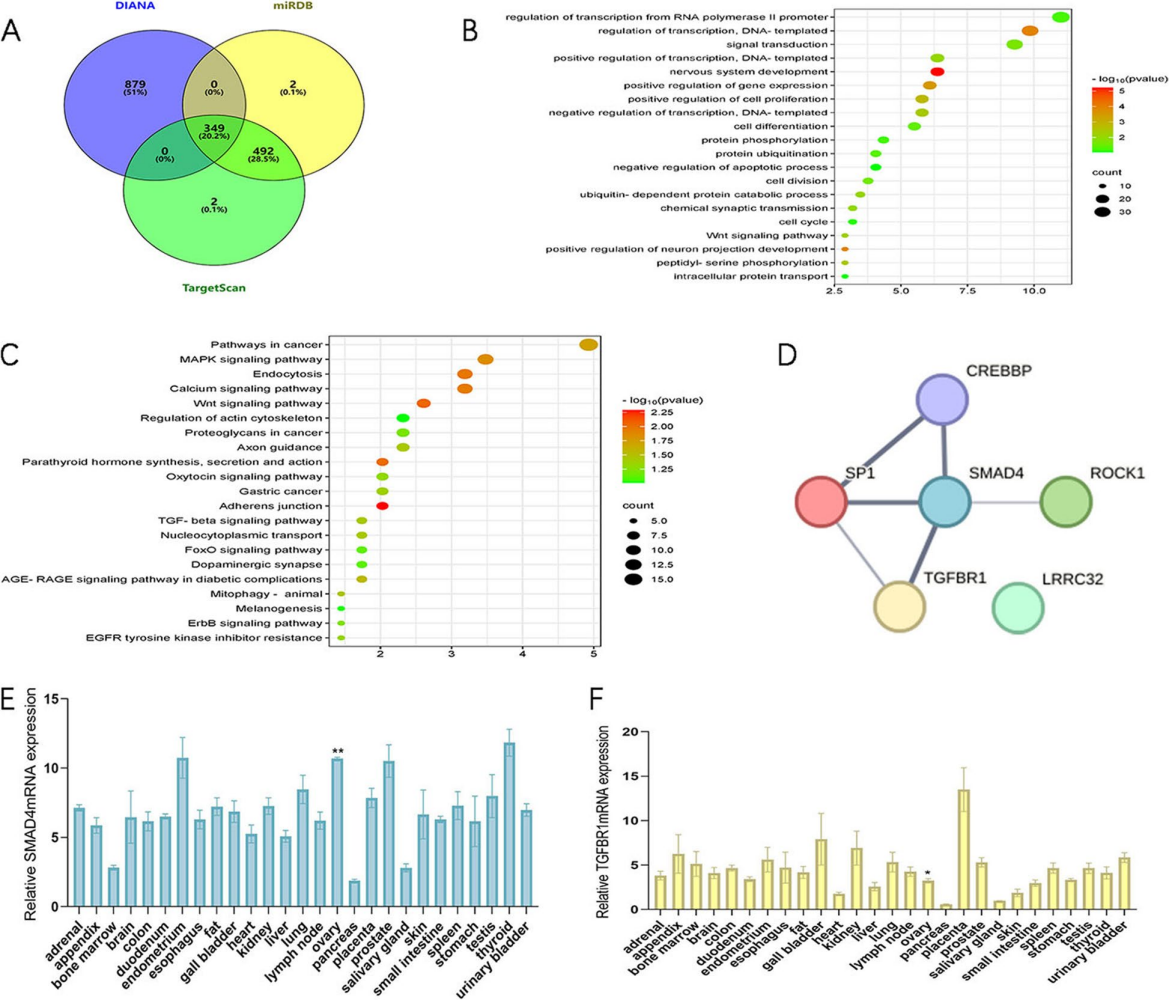
Potential effects of miR-6881-3p on ovarian granulosa cells on a transcriptome-wide scale and target gene screening. **A** Predicted targets of miR-6881-3p according to 3 databases; **B** Biological function analysis of 349 possible target genes; **C** KEGG analysis of 349 possible target genes; **D** Interactions between 6 proteins from the TGF-β signaling pathway; **E** Expression levels of SMAD4 mRNA in human tissues; **F** Expression levels of TGFBR1 mRNA in human tissues. **P < 0.01, *P < 0.05

A total of 349 potential target genes of miR‐6881‐3p were identified and are depicted in Table S1. The Protein-Protein Interaction (PPI) networks of 349 potential target genes are shown in Fig. S1. Subsequently, these 349 potential target genes underwent GO and KEGG analysis using the DAVID database. The biological function analysis highlighted enrichment in terms related to positive regulation of transcription, positive regulation of cell proliferation, and negative regulation of apoptotic processes (Fig. 2B), suggesting that miR-6881-3p may contribute to DOR by modulating cell proliferation or apoptosis. Additionally, the signaling pathway analysis indicated the TGF-β pathway as a potential candidate pathway (Fig. 2C). The interactions between 6 proteins from the TGF-β signaling pathway are shown in Fig. 2D. Only two target genes, SMAD4 and TGFBR1, associated with apoptosis were selected as candidates [11, 16, 17]. After querying the NCBI database, we discovered that SMAD4 mRNA expression levels were significantly higher in ovarian tissue compared to other tissues (*P* < 0.01), while TGFBR1 mRNA expression levels were relatively lower (*P* < 0.05).

### SMAD4 gene is a direct target of miR‐6881‐3p but not TGFBR1

**Fig. 3:**
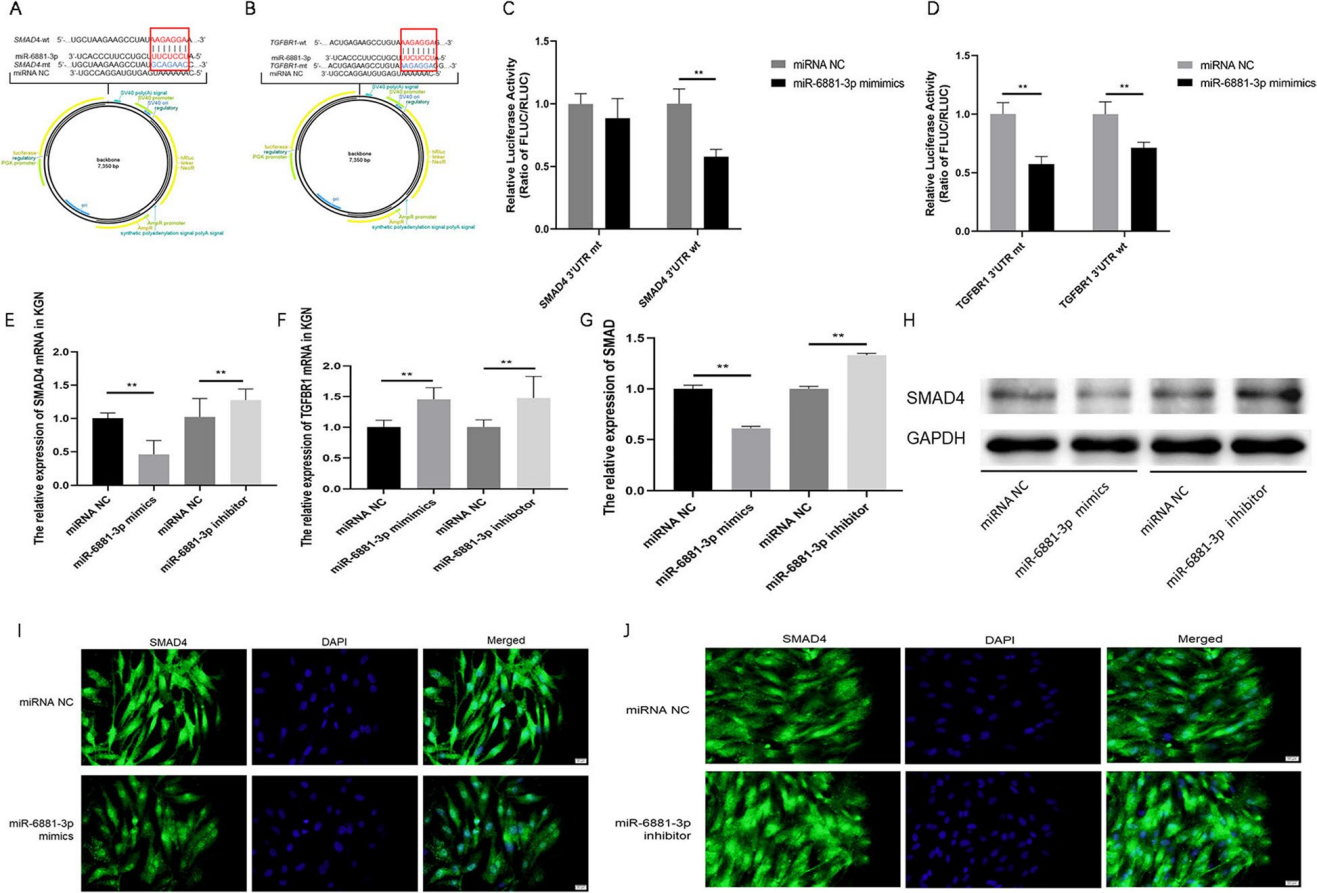
The SMAD4 gene is a downstream target of miR‐6881‐3p but not TGFBR1. **A**, **B** The miR-6881-3p binding site and mutation sites in the 3’-UTR of SMAD4 or TGFBR1; **C** The luciferase reporter assay showed direct binding between miR-6881-3p and the SMAD4 3’-UTR; **D** The luciferase reporter assay showed indirect binding between miR-6881-3p and the SMAD4 3’-UTR; **E** qRT‐PCR analysis of SMAD4 mRNA expression after transfection in KGN cell; **F** qRT‐PCR analysis of TGFBR1 mRNA expression after transfection in KGN cell; **G**, **H** Western blot analysis of SMAD4 protein levels after transfection in KGN cell (The original image is shown in Figs. S2 and S3); **I**, **J** The expression of SMAD4 in KGN cells detected by immunofluorescence staining. **P < 0.01

The dual‐luciferase assay results revealed that miR‐6881‐3p mimics significantly reduced the relative luciferase activity in the presence of SMAD4 3’-UTR WT compared with the negative control (NC) (*P* < 0.01) (Fig. 3C). Conversely, when mutations occurred in the SMAD4 3’-UTR (SMAD4-MT) binding site region, the suppressive effect of miR‐68811‐3p mimics was abrogated (Fig. 3C). However, miR-6881-3p mimics significantly reduced the relative luciferase activity in the presence of both TGFBR1 3’-UTR-WT and TGFBR1 3’-UTR-MT (*P* < 0.01), indicating that miR-6881-3p could not directly bind to the 3’-UTR of TGFBR1 (Fig. 3D).

Furthermore, we assessed the impact of miR-6881-3p on the expression of SMAD4 and TGFBR1 in KGN cells. Synthetic miR-6881-3p mimics/inhibitor were transfected into KGN cells. The results demonstrated that SMAD4 was downregulated in the miR-6881-3p mimics transfection group and upregulated in the miR-6881-3p inhibitor transfection group (Fig. 3E/G/H/I/J). However, the relative expression of TGFBR1 mRNA was upregulated in both the miR-6881-3p mimics and inhibitor transfection groups (*P* < 0.01) (Fig. 3F). These findings confirmed that miR-6881-3p can directly bind to SMAD4 mRNA in KGN cells and negatively regulate its transcription.

### miR-6881-3p promotes the apoptosis of KGN cells

**Fig. 4:**
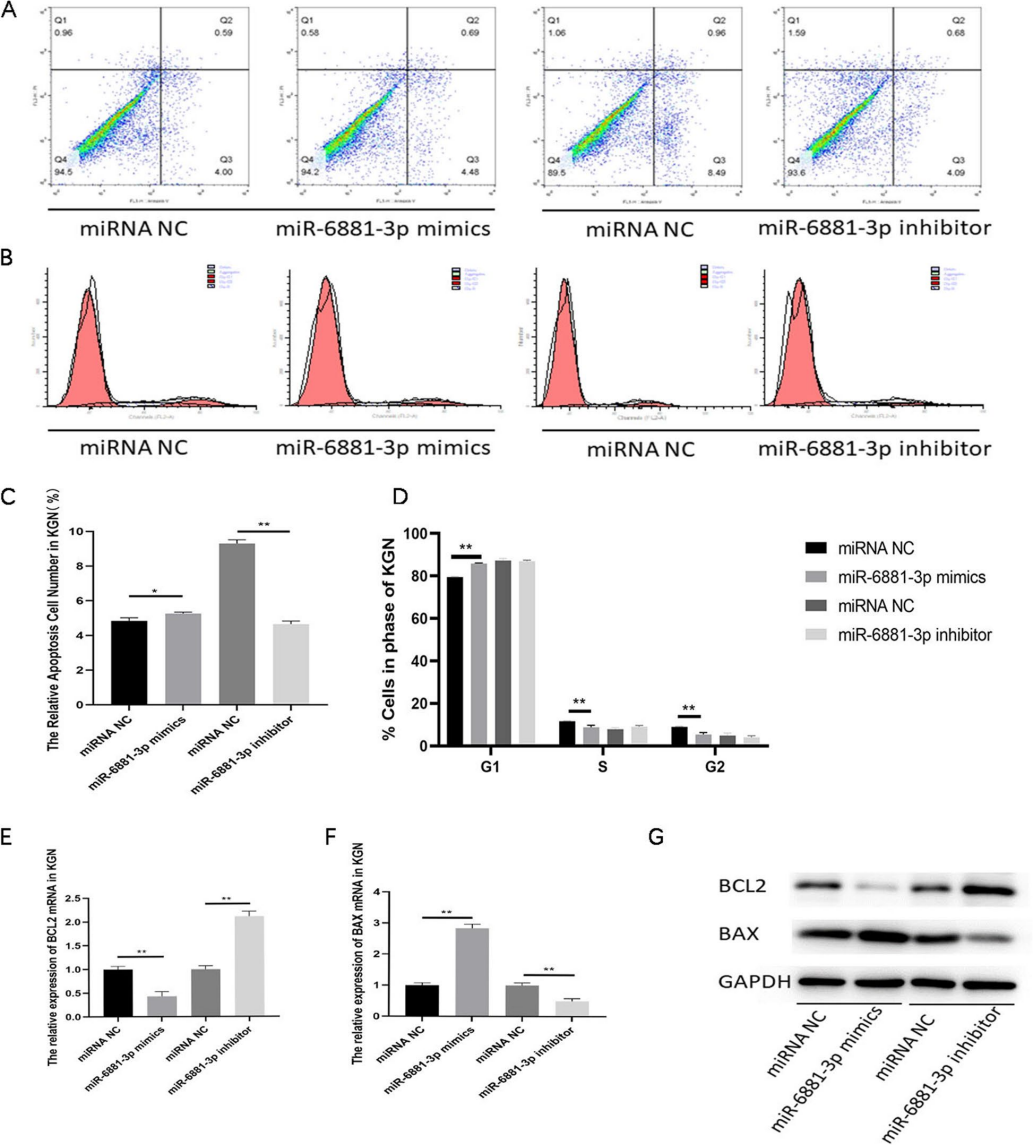
The effects of miR‐6881‐3p on KGN cell apoptosis. **A** Representative images of cell apoptosis in KGN cells after transfection; **B** Histogram representation of apoptotic rate in KGN cells; **C** Representative images of cell cycle in KGN cells after transfection; **D** Histogram representation of cell cycle rate in KGN cells; **E** qRT‐PCR analysis of BCL2 mRNA expression after transfection in KGN cell; **F** qRT‐PCR analysis of BAX mRNA expression after transfection in KGN cell; **G** Western blot analysis of BCL2 and BAX protein levels after transfection in KGN cell (The original image is shown in Figs. S4, S5 and S6) *P<0.05, **P<0.01

We subsequently investigated the effects of miR-6881-3p on GC function via flow cytometry. The results showed that miR-6881-3p mimics significantly reduced the percentage of viable cells and increased the percentage of early and late apoptotic cells (*P* < 0.05). In contrast, the opposite trend was observed in the miR-6881-3p inhibitor group (*P* < 0.01) (Fig. 4A-B). A significant increase in the number of cells in the G1 phase and a significant decrease in the number of cells in the S or G2 phase were detected in the miR-6881-3p mimics group of KGN cell, indicating that overexpression of miR-6881-3p increased the population of cells in the G1 phase (Fig. 4C-D). However, no significant differences in cell cycle progression were observed in the miR-6881-3p inhibitor transfection group of KGN cell (*P* > 0.05). In additon, BCL2 mRNA was downregulated in the miR-6881-3p mimics transfection group and upregulated in the miR-6881-3p inhibitor transfection group (Fig. 4E) (*P* < 0.01). However, the relative expression of BAX mRNA upregulated in the miR-6881-3p mimics transfection group and downregulated in the miR-6881-3p inhibitor transfection group (Fig. 4F) (*P* < 0.01). The expression levels of BCL2 and BAX proteins also showed differences after transfection in KGN cell (Fig. 4G). These results suggest that miR-6881-3p promotes the apoptosis of KGN cells.

### The expression levels of SMAD4 in human follicular fluid GCs are negatively correlated with the expression levels of miR-6881-3p and are associated with ovarian reserve and ART outcomes

**Fig. 5:**
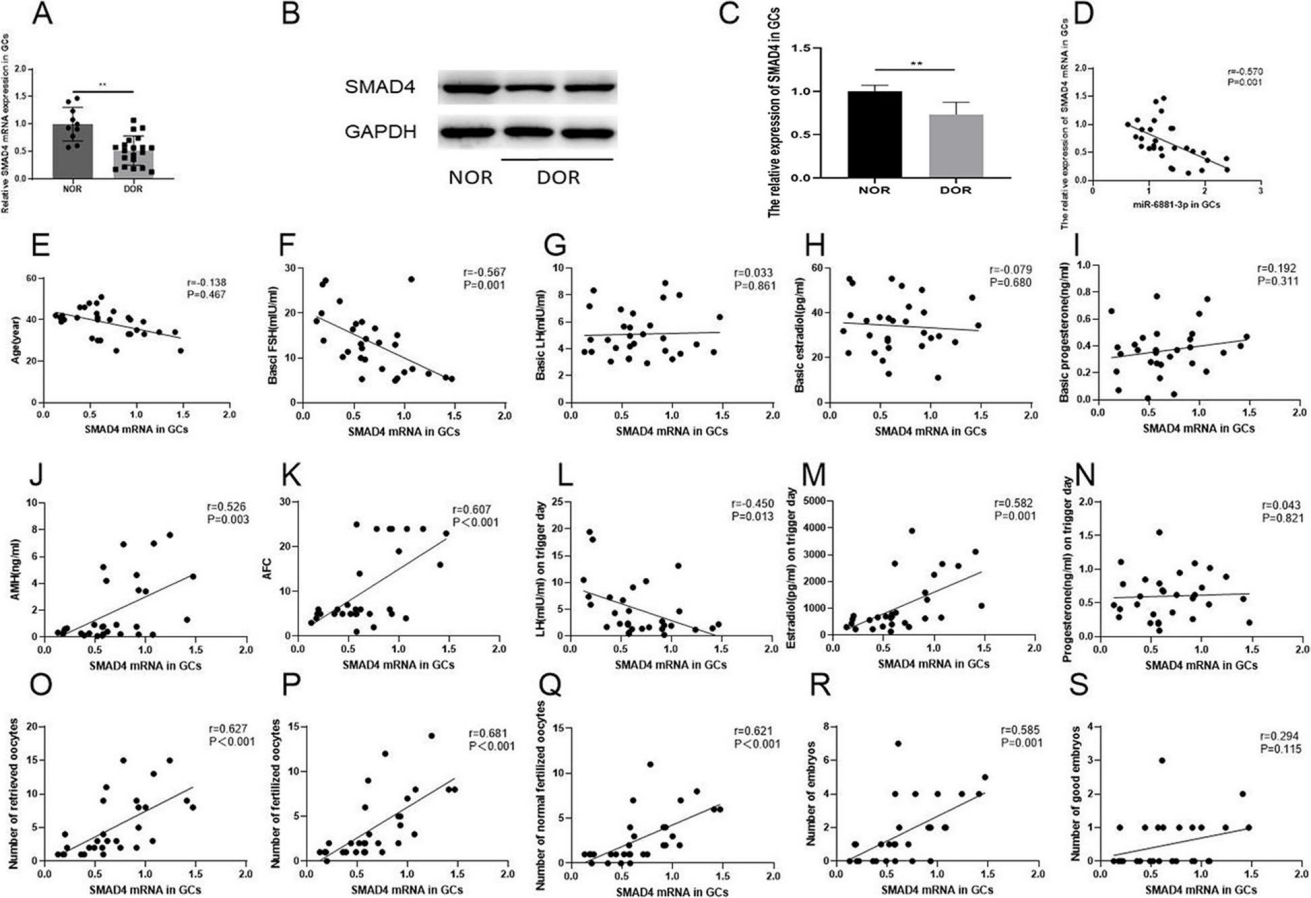
The expression levels of SMAD4 in human follicular fluid GCs are associated with ovarian reserve and ART outcomes. **A**-**C** The expression levels of SMAD4 of GCs in the NOR and DOR groups (The original image of WB is shown in Figs. S7 and S8); **D** Negative correlation between the expression levels of miR-6881-3p and SMAD4 mRNA in GCs; **E**-**K** Correlations between the expression levels of SMAD4 mRNA in GCs and age, basic FSH, basic LH, basic estradiol, basic progesterone, AMH, and AFC; **L**-**S** Correlations between the expression levels of SMAD4 mRNA in GCs and LH on trigger day, estradiol on trigger day, progesterone on trigger day, number of retrieved oocytes, number of fertilized oocytes, number of normal fertilized oocytes, number of embryos, and number of good embryos. ** *P* < 0.01

Further analysis of the follicular fluid GCs samples (*n* = 30) revealed that the expression levels of SMAD4 mRNA and proteins in the DOR group were significantly lower than those in the NOR group (*P* < 0.01, Fig. 5A-C). In addition, SMAD4 mRNA expression levels in GCs from human follicular fluid showed a significant negative correlation with miR-6881-3p expression levels (*r* = -0.570, *P* = 0.001) (Fig. 5D). A significant negative correlation was observed between SMAD4 mRNA levels in GCs from human follicular fluid and basal FSH (*r* = -0.567, *P* = 0.001) or LH on the trigger day (*r* = -0.450, *P* = 0.013), but a positive correlation with AMH (*r* = 0.526, *P* = 0.003), AFC (*r* = 0.607, *P* < 0.001), estradiol on trigger day (*r* = 0.582, *P* = 0.001), number of retrieved oocytes (*r* = 0.627, *P* < 0.001), number of fertilized oocytes (*r* = 0.681, *P* < 0.001), number of normal fertilized oocytes (*r* = 0.621, *P* < 0.001), and number of embryos (*r* = 0.585, *P* < 0.001). There were no significant correlations between SMAD4 mRNA levels and age, basal LH, basal estradiol, basal progesterone, progesterone on the trigger day, or the number of good embryos (all *P* > 0.05) (Fig. 5E-S).

### Increased expression of apoptotic proteins in follicular fluid GCs in DOR patients

**Fig. 6:**
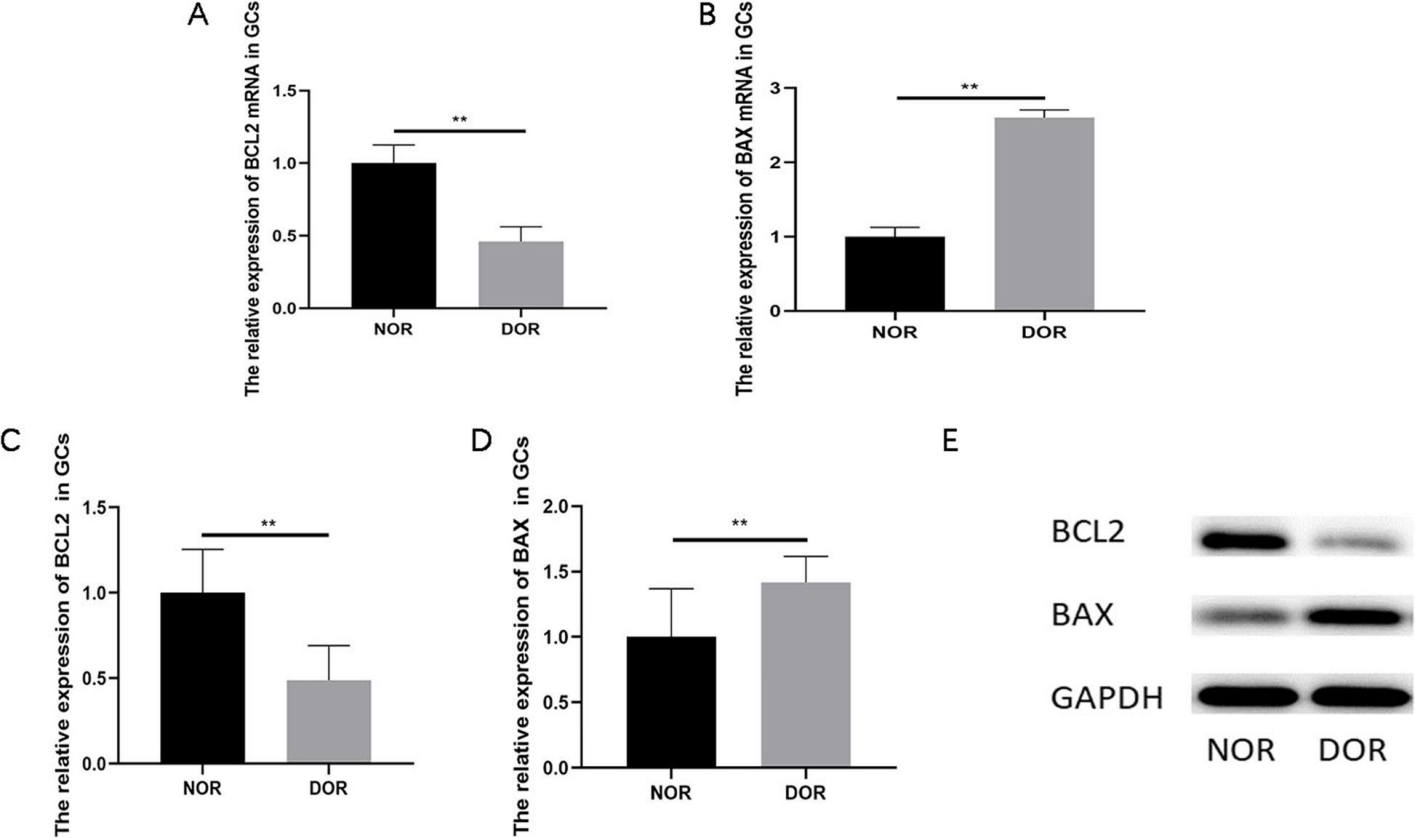
The expression levels of apoptosis related protein in human follicular fluid GCs. **A** qRT‐PCR analysis of BCL2 mRNA expression of GCs in the NOR group and DOR group; **B** qRT‐PCR analysis of BAX mRNA expression of GCs in the NOR group and DOR group; **C** Western blot analysis of BCL2 expression of GCs in the NOR group and DOR group; **D** Western blot analysis of BAX expression of GCs in the NOR group and DOR group; **E** Western blot analysis of BCL2 and BAX expression of GCs in the NOR group and DOR group (The original image is shown in Figs. S9, S10 and S11). ** *P* < 0.01

To further demonstrate that granulosa cells in follicular fluid of DOR patients are prone to transitional cell apoptosis, we detected the expression of apoptosis related proteins. Results show that the expression levels of BCL2 mRNA and proteins in the DOR group were significantly lower than those in the NOR group (*P* < 0.01, Fig. 6A/C/E). On the contrary, BAX mRNA and proteins in the DOR group were significantly higher than those in the NOR group (*P* < 0.01, Fig. 6B/D/E). The above results reveal that there may be excessive apoptosis in follicular fluid GCs of DOR patients.

### Differential expression of miR-6881-3p may affect regulation of gonadotropin receptors and steroid hormone generating enzyme in KGN cell and human follicular fluid GCs

**Fig. 7:**
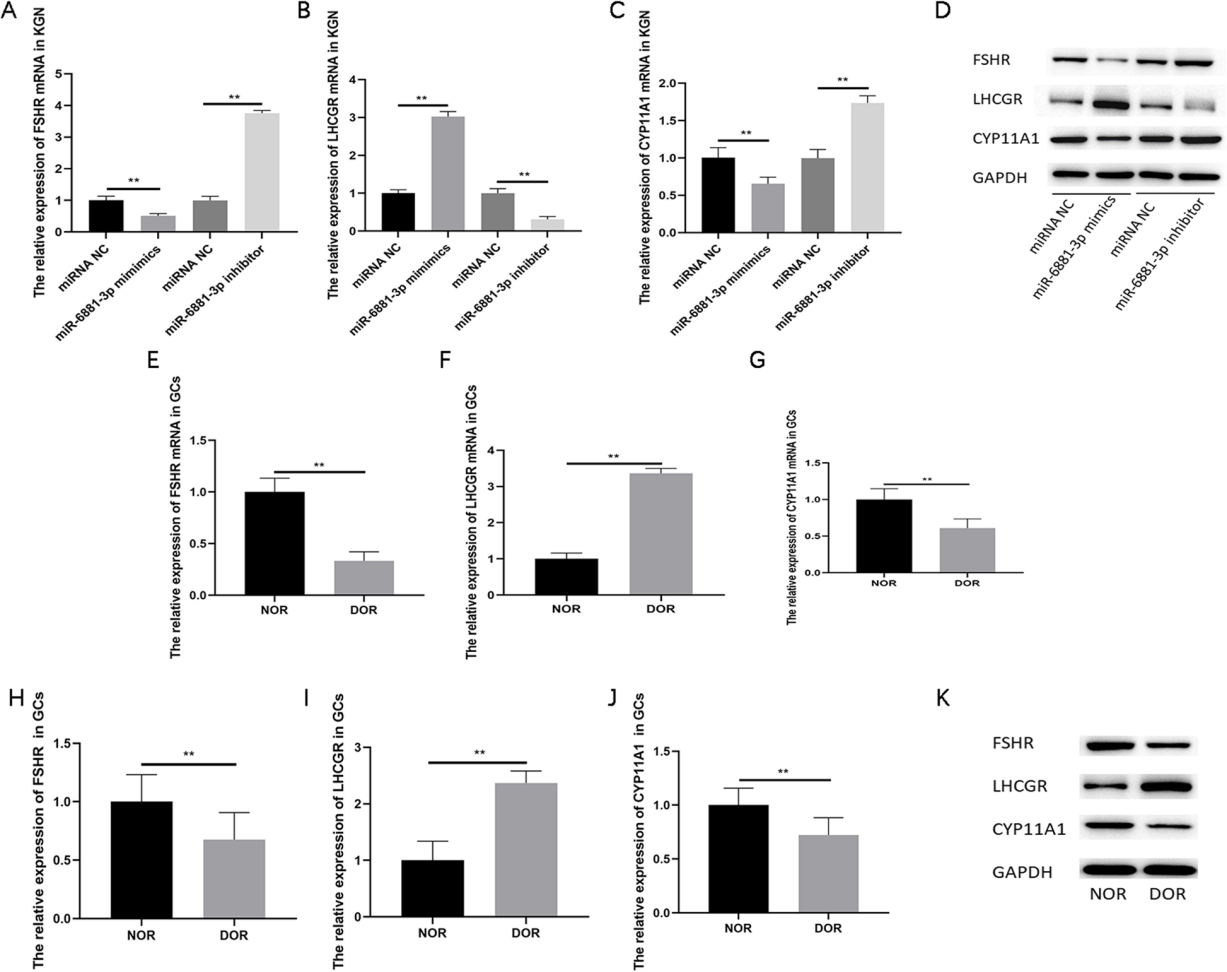
Differential regulation of gonadotropin receptors and steroid hormone generating enzyme in KGN cell and human follicular fluid GCs. **A** qRT‐PCR analysis of FSHR mRNA expression after transfection in KGN cell; **B** qRT‐PCR analysis of LHCGR mRNA expression after transfection in KGN cell; **C** qRT‐PCR analysis of CYP11A1 mRNA expression after transfection in KGN cell; **D** Western blot analysis of FSHR, LHCGR, CYP11A1 expression after transfection (The original image of WB is shown in Figs. S12, S13 and S14); **E** qRT‐PCR analysis of FSHR mRNA expression in the NOR and DOR groups; **F** qRT‐PCR analysis of LHCGR mRNA expression in the NOR and DOR groups; **G** qRT‐PCR analysis of CYP11A1 mRNA expression in the NOR and DOR groups; **H**-**K** Western blot analysis of FSHR, LHCGR, CYP11A1 proteins in the NOR and DOR group (The original image of WB is shown in Figs. S15, S16 and S17). ** *P* < 0.01

In order to better explain the physiological effects of miR-6881-3p on GCs, we detected the expression levels of gonadotropin receptors FSH receptor (FSHR), luteinizing hormone/choriogonadotropin receptor (LHCGR), and cholesterol side chain lyase (CYP11A1), a rate-limiting enzyme for steroid hormone synthesis. The results of KGN cells’ experiment demonstrated that FSHR and CYP11A1 mRNA were downregulated in the miR-6881-3p mimics transfection group and upregulated in the miR-6881-3p inhibitor transfection group (*P* < 0.01, Fig. 7A/C). However, the relative expression of LHCGR mRNA were upregulated in the miR-6881-3p mimics transfection group and downregulated in the miR-6881-3p inhibitor transfection group (*P* < 0.01, Fig. 7B). In human follicular fluid GCs, the expression levels of FSHR and CYP11A1 in the DOR group were significantly lower than those in the NOR group (*P* < 0.01, Fig. 7E/G/H/J/K), while the expression levels of LHCGR in the DOR group were significantly higher than those in the NOR group (*P* < 0.01, Fig. 7F/I/K). The above results indicate that the effect of miR-6881-3p on granulosa cells is not limited to cell apoptosis, but may further affect the differential expression of gonadotropin receptors and regulate steroid hormone synthesis.

## Discussion

It is now understood that maintaining good-quality mature oocytes is essential for female fertility. Indeed, ovarian GCs are pivotal in nurturing oocytes through gap junctions and releasing paracrine signals that regulate oocyte development [4]. The intricate interaction between oocytes and surrounding GCs is critical for follicular development and producing fertilizable oocytes [18]. Dysfunctions in GCs may significantly contribute to DOR [19, 20]. There is an increasing consensus suggesting that miRNA dysregulation is associated with various reproductive conditions, such as DOR [21], premature ovarian failure (POF) [22], and polycystic ovary syndrome (PCOS) [23]. In this respect, miR-484 levels in GCs from human follicular fluid have been linked to ovarian reserve, with miR-484 promoting mitochondrial damage and GC apoptosis in DOR patients by targeting YAP1 [9]. Additionally, downregulation of miR-106a expression may contribute to DOR pathogenesis by enhancing ASK1 signaling and promoting GC apoptosis [24].

Our study revealed a negative correlation between miR-6881-3p expression levels and female ovarian function, with higher expression observed in the DOR group. Notably, miR-6881-3p levels in GCs from human follicular fluid exhibited a significant positive correlation with basic FSH levels but a negative correlation with AMH and AFC. Currently, AMH is considered the gold standard for assessing ovarian function, as it is secreted by GCs and reflects the number of primary follicles and their ability to regulate follicle maturation and development [25]. AFC is a reliable indicator of ovarian reserve in women of childbearing age and is closely linked to ovarian response during the ovulation cycle [26]. Clinical observations have indicated that basal FSH levels increase with age and ovarian insufficiency, often necessitating higher FSH stimulation to initiate follicular development [27, 28]. In IVF/ICSI cycles, miR-6881-3p expression levels in GCs from human follicular fluid were negatively correlated with the number of retrieved oocytes and embryos. These findings suggest that miR-6881-3p may be closely associated with ovarian function, warranting further research into its role in DOR.

Subsequent experiments revealed that miR-6881-3p induces apoptosis in KGN cell. Through bioinformatics prediction, luciferase reporter assays, qRT-PCR, and Western blot analysis, we identified SMAD4 as a direct target of miR-6881-3p. It is well-established that SMAD4 is a central component of the canonical TGF-β signaling pathway. Given the widespread expression of TGF-β and its receptors in the reproductive system [29, 30]. SMAD4 is likely a key mediator in regulating reproductive function. For instance, TGF-β1 induces TEX14-IT1 transcription in a SMAD4-dependent manner, and TEX14-IT1 mediates the antiapoptotic and pro-proliferative effects of TGF-β1 in GCs [31]. SMAD4 is a key regulatory factor in the production of FSH [32, 33]. Bone morphogenetic protein 15 (BMP15) regulates FSH receptor (FSHR) through TGF-β receptor II and SMAD4 signaling in porcine GCs to regulate follicular development [34, 35]. Knockdown of SMAD4 significantly inhibited FSH-induced porcine GCs proliferation and estradiol production [36]. The present study further demonstrated that SMAD4 mRNA expression levels in GCs from human follicular fluid were positively correlated with ovarian reserve. Therefore, it is highly conceivable that the upregulation of miR-6881-3p may contribute to the pathogenesis of DOR by promoting GC apoptosis through the downregulation of SMAD4 expression.

It is worth noting that SMAD4’s regulatory function can be achieved post-transcriptionally through the SMAD4-binding element (SBE) within the gene’s promoter region [16, 17, 37]. Thus, SMAD4 serves as a negative transcriptional regulator in GCs. We further explored the binding relationship between SBE and the MIR6881 promoter region through bioinformatics prediction and dual‐luciferase reporter analysis, revealing that SMAD4 is not a transacting element that negatively regulates MIR6881 transcription (Fig. S18).

In addition, due to the close interaction between oocytes and granulosa cells during follicular development [38], it is necessary to investigate whether the physiological function of granulosa cells is affected in DOR. FSHR is G-protein coupled receptor, which is primarily located on granulosa cells in the ovary. After FSH specifically binds to FSHR, it activates aromatase and promotes the production of LH receptors. According to current research, polymorphisms have been identified within the FSHR gene that differed in frequency among patients with poor ovarian response and DOR [39, 40]. LHCGR are found on GCs after induction by FSH. LHCGR is important in ovarian steroidogenesis, luteinization, and therefore, progesterone synthesis and fertility. Skiadas et al. found a significantly increased expression of the LHCGR gene in DOR patients over NOR patients [41]. The biosynthesis of progesterone and estradiol in response to FSH involves the regulation of multiple steroidogenic enzymes, such as p450 cholesterol side-chain cleavage enzyme(CYP11A) and aromatase (CYP19), the markers of ovarian follicle proliferation and differentiation [42]. Our research is consistent with the above findings: the expression levels of FSHR and CYP11A1 in the DOR group were significantly lower than those in the NOR group, while the expression levels of LHCGR in the DOR group were significantly higher than those in the NOR group. Surprisingly, differential expression consistent with clinical phenotype was observed in KGN cells transfected with miR-6881-3p mimics or miR-6881-3p inhibitor. This means that the ability of follicular fluid GCs in DOR patients to receive gonadotropin stimulation and produce steroid hormones decreases, and may be a result of elevated miR-6881-3p levels, which deserves further research to determine.

In recent years, numerous studies have highlighted the importance of the miRNA-mRNA regulatory network in ovarian disease development, shedding light on potential mechanisms underlying DOR. MiRNA-based therapy, which regulates the expression of multiple genes, may offer a promising therapeutic strategy compared to traditional single-target therapy. However, this study has its limitations, notably the absence of in vivo experimental data. Culturing isolated GCs to enable additional analyses is needed for further study.

In summary, the upregulation of miR-6881-3p may contribute to the pathogenesis of DOR by promoting GC apoptosis via the downregulation of SMAD4 expression. Furthermore, we substantiated that high miR-6881-3p expression and low SMAD4 expression in DOR patients could serve as potential diagnostic markers and therapeutic targets for DOR.

## Conclusion

This study underscores the role of miR-6881-3p in directly targeting SMAD4 mRNA, subsequently diminishing granulosa cell viability and promoting apoptosis, and may affect steroid hormone regulation and gonadotropin signal reception in granulosa cells. These findings contribute to our understanding of the pathogenesis of DOR. However, more rigorous in vivo experiments are still needed to validate our findings.

### Supplementary Information

**Additional file 1: Supplementary Table 1.** 349 potential target genes of miR‐6881‐3p.

**Additional file 2: Figure S1.** The Protein-Protein Interaction (PPI) networks of 349 potential target genes (Genes with dark colors and large nodes indicate that they have more connections with other genes).

**Additional file 3: Figure S2.** The original image of Western blot analysis of SMAD4 protein levels after transfection in KGN cell (From left to right are miRNA NC group, miR-6881-3p mimics group, miRNA NC group, miR-6881-3p inhibitor group).

**Additional file 4: Figure S3.** The original image of Western blot analysis of GAPDH protein levels after transfection in KGN cell (From left to right are miRNA NC group, miR-6881-3p mimics group, miRNA NC group, miR-6881-3p inhibitor group).

**Additional file 5: Figure S4.** The original image of Western blot analysis of BCL2 protein levels after transfection in KGN cell (From left to right are miRNA NC group, miR-6881-3p mimics group, miRNA NC group, miR-6881-3p inhibitor group).

**Additional file 6: Figure S5.** The original image of Western blot analysis of BAX protein levels after transfection in KGN cell (From left to right are miRNA NC group, miR-6881-3p mimics group, miRNA NC group, miR-6881-3p inhibitor group).

**Additional file 7: Figure S6.** The original image of Western blot analysis of GAPDH protein levels after transfection in KGN cell (From left to right are miRNA NC group, miR-6881-3p mimics group, miRNA NC group, miR-6881-3p inhibitor group).

**Additional file 8: Figure S7.** The original image of Western blot analysis of SMAD4 protein levels in GCs (From left to right are NOR, DOR, DOR, repeat five times).

**Additional file 9: Figure S8.** The original image of Western blot analysis of GAPDH protein levels in GCs (From left to right are NOR, DOR, DOR, repeat five times).

**Additional file 10: Figure S9.** The original image of Western blot analysis of BCL2 protein levels in GCs (From left to right are NOR, DOR, repeat three times).

**Additional file 11: Figure S10.** The original image of Western blot analysis of BAX protein levels in GCs (From left to right are NOR, DOR, repeat three times).

**Additional file 12: Figure S11.** The original image of Western blot analysis of GAPDH protein levels in GCs (From left to right are NOR, DOR, repeat three times).

**Additional file 13: Figure S12.** The original image of Western blot analysis of FSHR protein levels after transfection in KGN cell (From left to right are miRNA NC group, miR-6881-3p mimics group, miRNA NC group, miR-6881-3p inhibitor group).

**Additional file 14: Figure S13.** The original image of Western blot analysis of LHCGR protein levels after transfection in KGN cell (From left to right are miRNA NC group, miR-6881-3p mimics group, miRNA NC group, miR-6881-3p inhibitor group).

**Additional file 15: Figure S14.** The original image of Western blot analysis of CYP11A1 protein levels after transfection in KGN cell (From left to right are miRNA NC group, miR-6881-3p mimics group, miRNA NC group, miR-6881-3p inhibitor group).

**Additional file 16: Figure S15.** The original image of Western blot analysis of FSHR protein levels in GCs (From left to right are NOR, DOR, repeat three times).

**Additional file 17: Figure S16.** The original image of Western blot analysis of LHCGR protein levels in GCs (From left to right are NOR, DOR, repeat three times).

**Additional file 18: Figure S17.** The original image of Western blot analysis of CYP11A1 protein levels in GCs (From left to right are NOR, DOR, repeat three times).

**Additional file 19: Figure S18.** The luciferase reporter assay of SMAD4-binding element (SBE) and MIR6881 gene promoter (There are no significant differences in the relative luciferase activity in the presence of both MIR6881 promter-WT and MIR6881 promoter-MT, indicating that SBE could not directly bind to the MIR6881 promoter).

## Data Availability

All data generated or analysed during this study are included in this published article and its supplementary information files, more detailed datas are available from the respective authors (Shan Xiang: axiangshan@163.com or Fang Lian: lianfangbangong@163.com) upon reasonable request.

## Abbreviations

AMH: Antimullerian hormone
ART: Assisted reproductive technology
BMI: Body mass index
ICSI: Intracytoplasmic sperm injection
FSH: Follicle-stimulating hormone
LH: Luteinizing hormone
IVF: In vitro fertilization
MT: Mutation-type
UTR: Untranslated region
WT: Wild-type
