# Supplementary figures and images for: miR-6881-3p contributes to diminished ovarian reserve by regulating granulosa cell apoptosis by targeting SMAD4
Source: Reprod Biol Endocrinol. 2024 Feb 1;22:17. doi: 10.1186/s12958-024-01189-8 (PMC10832098; doi:10.1186/s12958-024-01189-8)

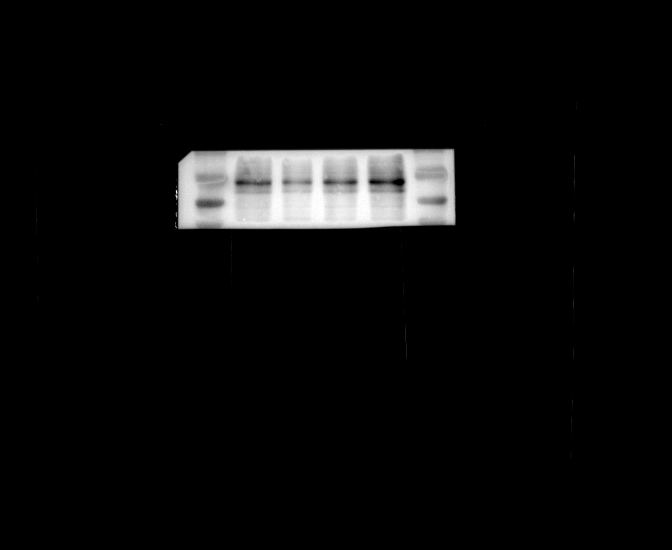

Supplement: Supplementary file 3 — Additional file 3: Figure S2. The original image of Western blot analysis of SMAD4 protein levels after transfection in KGN cell (From left to right are miRNA NC group, miR-6881-3p mimics group, miRNA NC group, miR-6881-3p inhibitor group). [file 12958_2024_1189_MOESM3_ESM.tif]

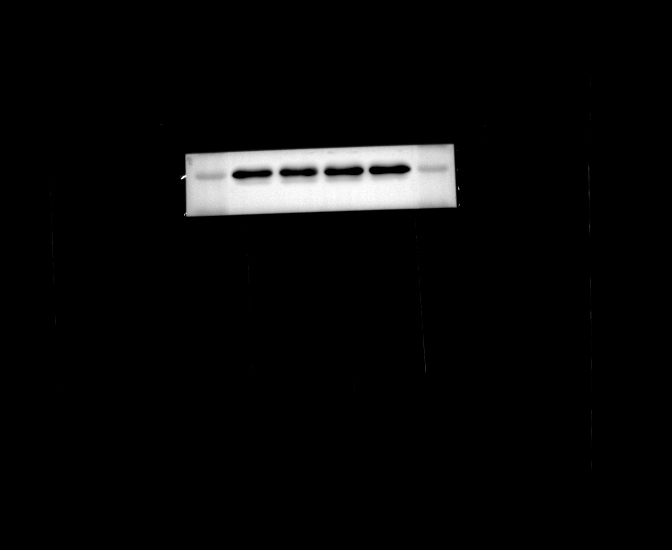

Supplement: Supplementary file 4 — Additional file 4: Figure S3. The original image of Western blot analysis of GAPDH protein levels after transfection in KGN cell (From left to right are miRNA NC group, miR-6881-3p mimics group, miRNA NC group, miR-6881-3p inhibitor group). [file 12958_2024_1189_MOESM4_ESM.tif]

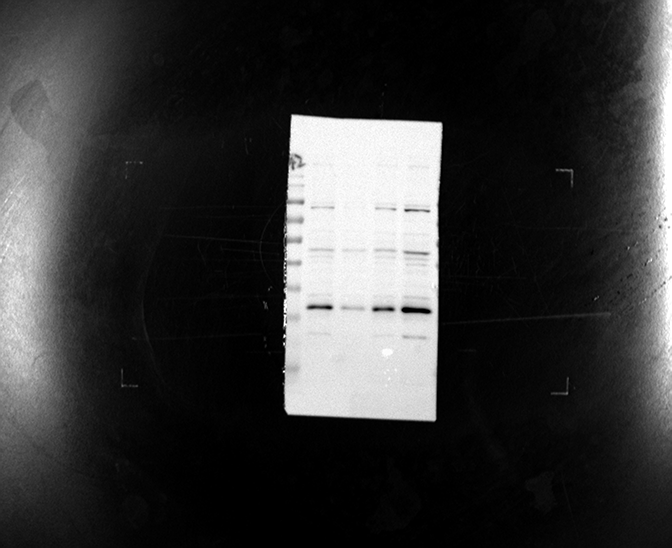

Supplement: Supplementary file 5 — Additional file 5: Figure S4. The original image of Western blot analysis of BCL2 protein levels after transfection in KGN cell (From left to right are miRNA NC group, miR-6881-3p mimics group, miRNA NC group, miR-6881-3p inhibitor group). [file 12958_2024_1189_MOESM5_ESM.tif]

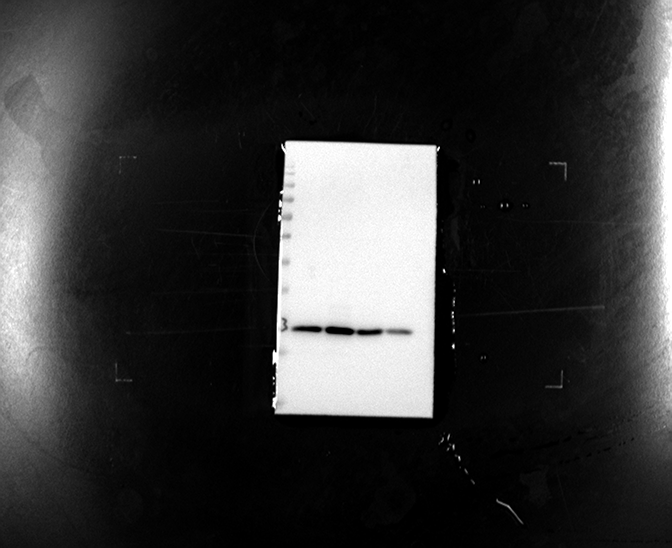

Supplement: Supplementary file 6 — Additional file 6: Figure S5. The original image of Western blot analysis of BAX protein levels after transfection in KGN cell (From left to right are miRNA NC group, miR-6881-3p mimics group, miRNA NC group, miR-6881-3p inhibitor group). [file 12958_2024_1189_MOESM6_ESM.tif]

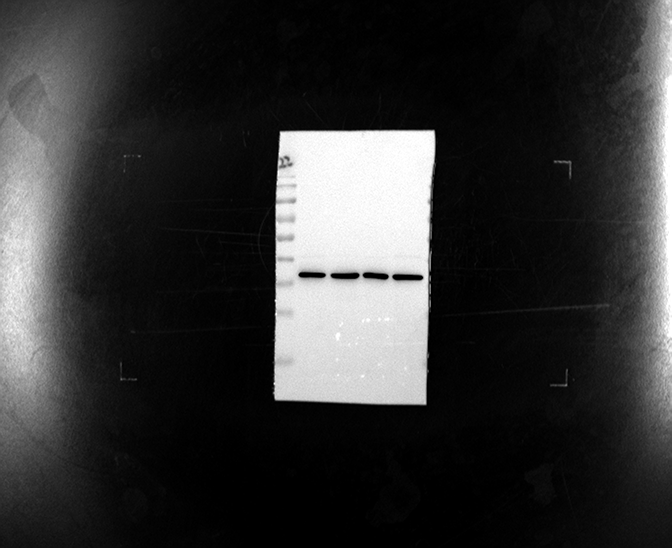

Supplement: Supplementary file 7 — Additional file 7: Figure S6. The original image of Western blot analysis of GAPDH protein levels after transfection in KGN cell (From left to right are miRNA NC group, miR-6881-3p mimics group, miRNA NC group, miR-6881-3p inhibitor group). [file 12958_2024_1189_MOESM7_ESM.tif]

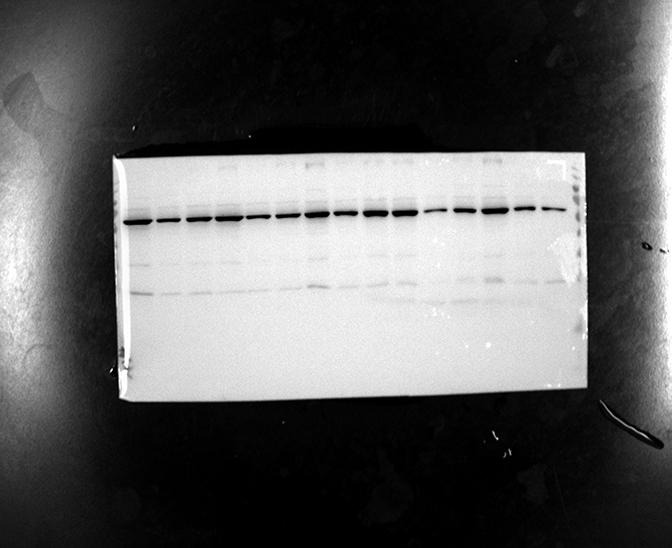

Supplement: Supplementary file 8 — Additional file 8: Figure S7. The original image of Western blot analysis of SMAD4 protein levels in GCs (From left to right are NOR, DOR, DOR, repeat five times). [file 12958_2024_1189_MOESM8_ESM.tif]

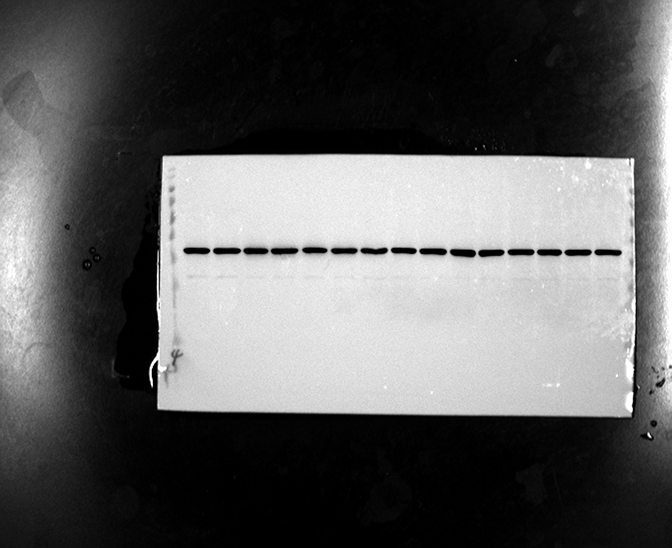

Supplement: Supplementary file 9 — Additional file 9: Figure S8. The original image of Western blot analysis of GAPDH protein levels in GCs (From left to right are NOR, DOR, DOR, repeat five times). [file 12958_2024_1189_MOESM9_ESM.tif]

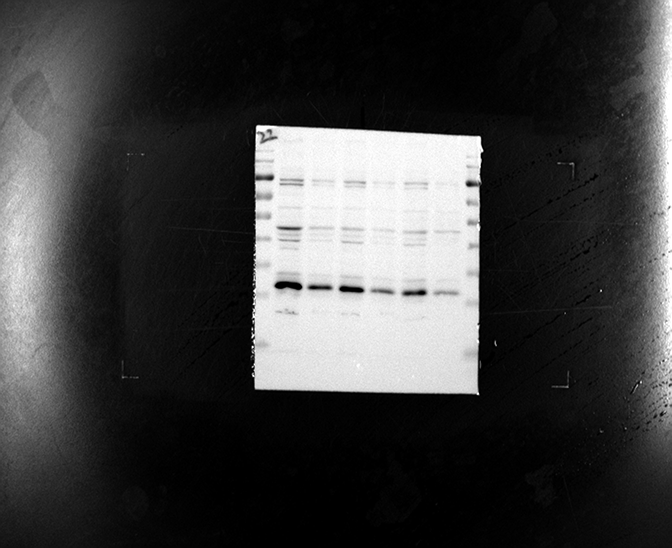

Supplement: Supplementary file 10 — Additional file 10: Figure S9. The original image of Western blot analysis of BCL2 protein levels in GCs (From left to right are NOR, DOR, repeat three times). [file 12958_2024_1189_MOESM10_ESM.tif]

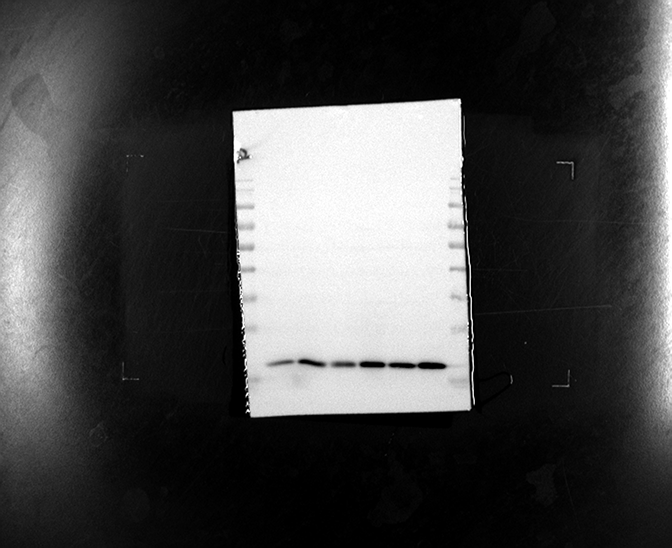

Supplement: Supplementary file 11 — Additional file 11: Figure S10. The original image of Western blot analysis of BAX protein levels in GCs (From left to right are NOR, DOR, repeat three times). [file 12958_2024_1189_MOESM11_ESM.tif]

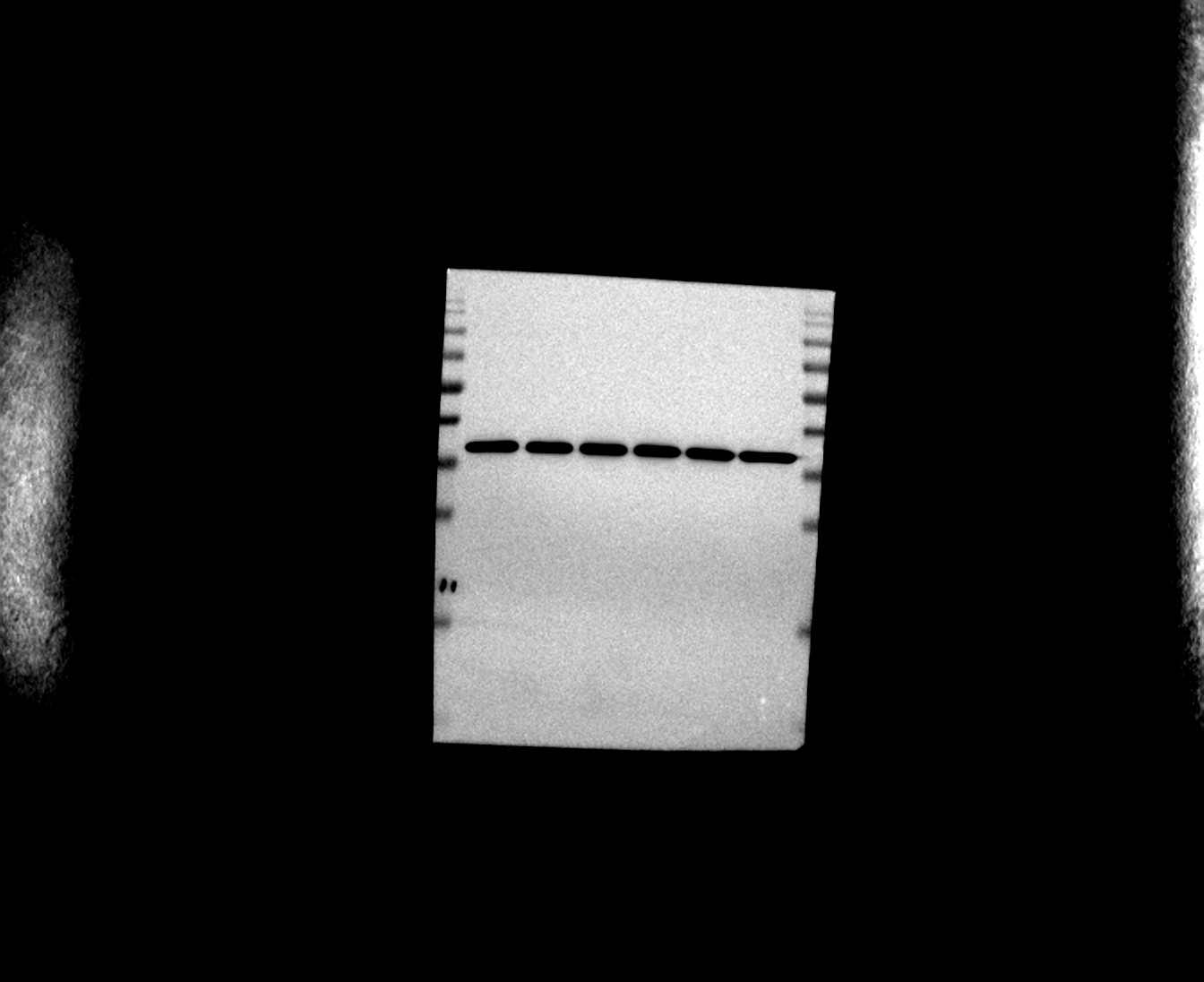

Supplement: Supplementary file 12 — Additional file 12: Figure S11. The original image of Western blot analysis of GAPDH protein levels in GCs (From left to right are NOR, DOR, repeat three times). [file 12958_2024_1189_MOESM12_ESM.tif]

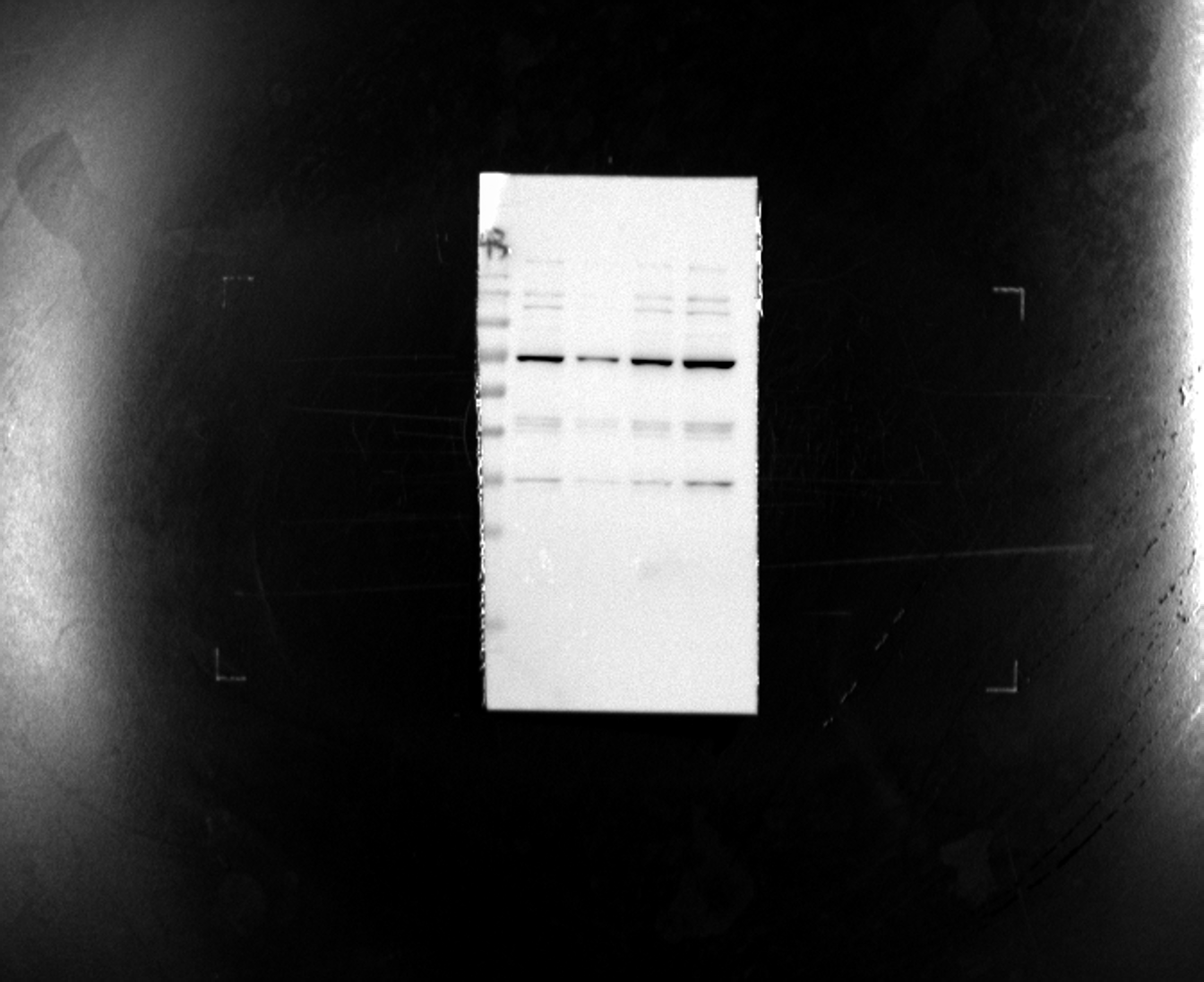

Supplement: Supplementary file 13 — Additional file 13: Figure S12. The original image of Western blot analysis of FSHR protein levels after transfection in KGN cell (From left to right are miRNA NC group, miR-6881-3p mimics group, miRNA NC group, miR-6881-3p inhibitor group). [file 12958_2024_1189_MOESM13_ESM.tif]

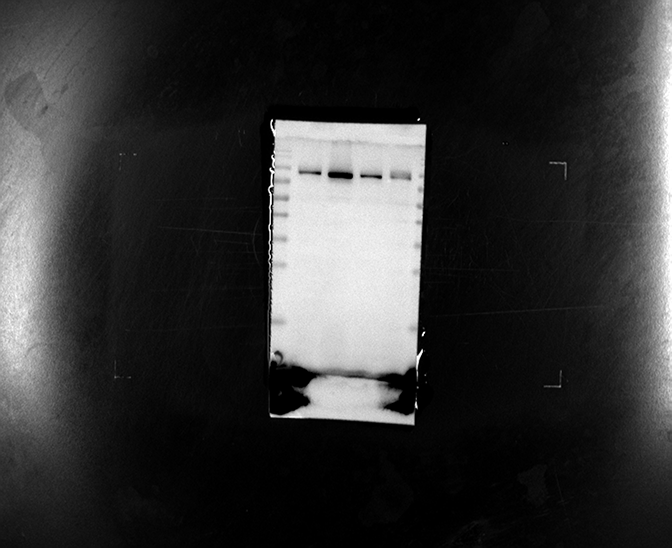

Supplement: Supplementary file 14 — Additional file 14: Figure S13. The original image of Western blot analysis of LHCGR protein levels after transfection in KGN cell (From left to right are miRNA NC group, miR-6881-3p mimics group, miRNA NC group, miR-6881-3p inhibitor group). [file 12958_2024_1189_MOESM14_ESM.tif]

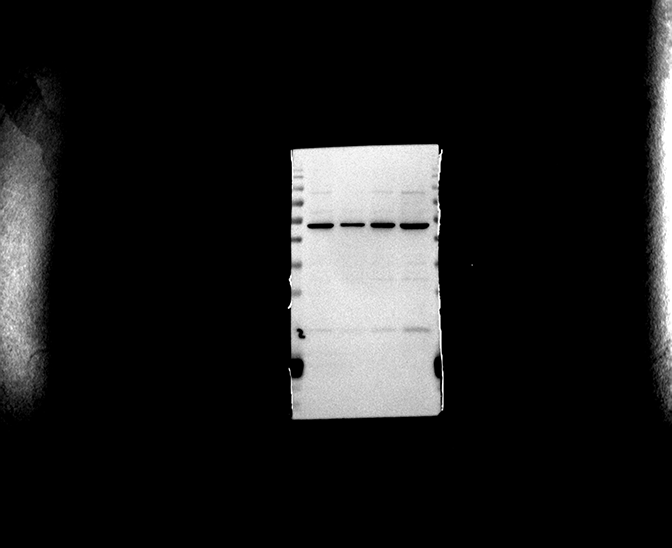

Supplement: Supplementary file 15 — Additional file 15: Figure S14. The original image of Western blot analysis of CYP11A1 protein levels after transfection in KGN cell (From left to right are miRNA NC group, miR-6881-3p mimics group, miRNA NC group, miR-6881-3p inhibitor group). [file 12958_2024_1189_MOESM15_ESM.tif]

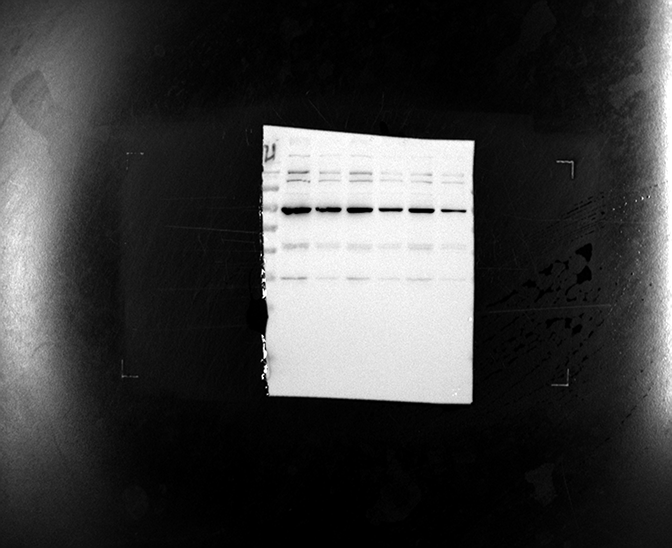

Supplement: Supplementary file 16 — Additional file 16: Figure S15. The original image of Western blot analysis of FSHR protein levels in GCs (From left to right are NOR, DOR, repeat three times). [file 12958_2024_1189_MOESM16_ESM.tif]

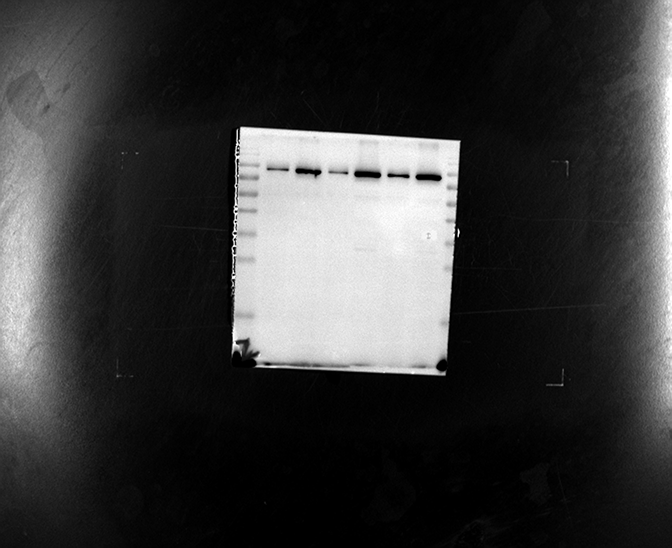

Supplement: Supplementary file 17 — Additional file 17: Figure S16. The original image of Western blot analysis of LHCGR protein levels in GCs (From left to right are NOR, DOR, repeat three times). [file 12958_2024_1189_MOESM17_ESM.tif]

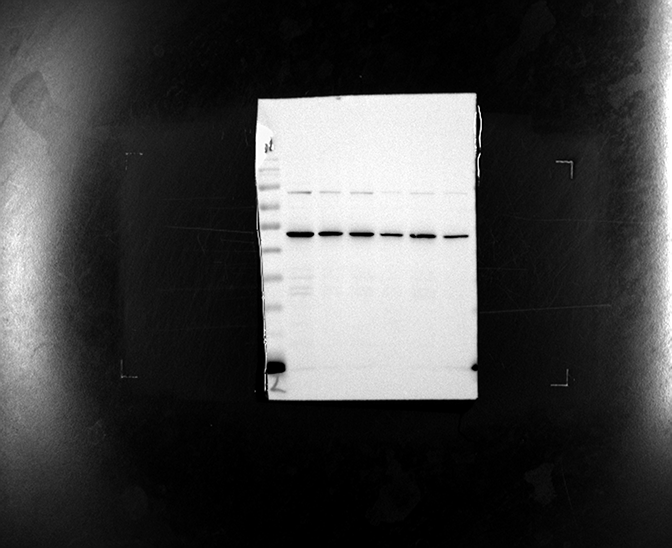

Supplement: Supplementary file 18 — Additional file 18: Figure S17. The original image of Western blot analysis of CYP11A1 protein levels in GCs (From left to right are NOR, DOR, repeat three times). [file 12958_2024_1189_MOESM18_ESM.tif]
